# *Justiciathailandica*, a new species of Acanthaceae from Thailand

**DOI:** 10.3897/phytokeys.124.33745

**Published:** 2019-06-06

**Authors:** Yi Tong, Yunfei Deng

**Affiliations:** 1 School of Chinese Materia Medica, Guangzhou University of Chinese Medicine, Guangzhou 510006, Guangdong, China Guangzhou University of Chinese Medicine Guangzhou China; 2 Key Lab of Plant Resources Conservation and Sustainable Utilization, South China Botanical Garden, Chinese Academy of Sciences, Guangzhou 510650, Guangdong, China South China Botanical Garden, Chinese Academy of Sciences Guangzhou China; 3 Southeast Asia Biodiversity Research Institute, Chinese Academy of Sciences, Yezin, Nay Pyi Taw 05282, Myanmar Southeast Asia Biodiversity Research Institute, Chinese Academy of Sciences Nay Pyi Taw Myanmar

**Keywords:** *
Harnieria
*, *
Calophanoides
*, new taxa, taxonomy

## Abstract

A new species of *Justicia* (Acanthaceae), *J.thailandica*, is described and illustrated from Thailand. The new species belongs to Justiciasect.Harnieria and is similar to *J.quadrifaria* and *J.championii*, but differs on account of the obviously densely white indumentum in the inflorescence bracts and calyx, ovate leaf blades with margin usually entire, spathulate inflorescence bracts and length ratio of calyx to mature capsule. It is assessed to be “Near threatened” (NE) according to IUCN Red List Category and Criteria. Pollen and seed morphology characters are also reported. Species of Justiciasect.Harnieria in Thailand are discussed and a key to the three recognized species is presented.

## Introduction

*Justicia* L. is the largest genus in the family Acanthaceae and consists of about 600 species distributed in tropical and temperate (to a lesser extent) regions of the world (Graham 1988; Hu et al. 2011; Mabberley 2017). It is characterized by the tubular and bilabiate corolla with stylar furrow (rugula) in the upper lip, two stamens usually with the lower anther-theca spurred at base, “Knötchenpollen’’ pollen grains, and 4-(rarely 2-)seeded stalked capsules (Lindau 1894; Graham 1988; Hu et al. 2011). The recent molecular evidence (Deng et al. 2016; Kiel et al. 2017) indicated that the genus *Justicia* in the broad sense is polyphyletic and might be further separated into several independent genera. At the moment, we follow the treatment of Graham (1988) who divided the genus into sixteen sections.

Sect.Harnieria (Solms-Laubach) Benth. is characterized by the abbreviated axillary spikes, leaf-like inflorescence bracts, fusiform capsules and tuberculate seeds (Tong et al. 2016). It comprises approximately 76 species distributed in the tropical and subtropical regions of Africa and Asia with two species extending to Australia (Barker 1986; Hedrén 1989; Tong et al. 2016).

In the course of revising Justiciasect.Harnieria from Asia, some specimens collected from Thailand and identified in herbaria as *J.quadrifaria* (Nees) T. Anderson or *J.championii* T. Anderson appear to represent an undescribed species, which differs from the latter two species by the characters of indumentum, leaves, petiole, calyx, inflorescence bracts and length ratio of calyx to mature capsule.

## Materials and methods

The morphological comparison with related species in Justiciasect.Harnieria was based on studies of herbarium specimens and information gathered from literature. Pollen grains and seeds were taken from dried specimens (Beusekom *et al.* 3759, MO2366671) and mounted on aluminium stubs coated with gold in a sputter coater after being cleaned in water using ultrasound, and then examined using scanning electron microcopy (SEM; JSM-6360LV). The polar (P) axis and equatorial (E) diameter were measured by imaging analyzer (Smile View 2.1; JEOL Tokyo, Japan). Pollen terminology follows Erdtman (1969) and Punt et al. (2007). Seed terminology follows Hedrén (1989) and Rueangsawang et al. (2012).

## Taxonomic description

### Justicia thailandica

Taxon classificationPlantaeLamialesAcanthaceae

Y.F.Deng & Y.Tong sp. nov.

urn:lsid:ipni.org:names:77197854-1

Figures 1 , 2

#### Type.

THAILAND. Kanchanaburi Province, Kanchanaburi District, Huay Bankau, 14°55'00"N, 98°45'00"E, mixed deciduous forest on limestone, 900 m alt., 13 Nov 1971, C. F. van Beusekom, C. Phengklai, R. Geesink & B. Wongwan 3759 (holotype: MO2366671!; isotypes: BKF!, C!, K!, L!, P!).

#### Diagnosis.

The new species is similar to *Justiciaquadrifaria* (Nees) T. Anderson, but differs on account of the whole plant being white villous (not pubescent), leaf blade ovate (not oval, oblong to rarely ovate) with margin usually entire (not slightly undulate), apex shortly caudate or acute (not acuminate), base cuneate (not decurrent), petiole 8−12 mm (not 5−7 mm) long, calyx densely white villous (not pubescent), inflorescence bracts spathulate and villous (not ovate to obovate and sparsely pubescent) and capsule longer than the calyx (not shorter than the calyx). It is also similar to *J.championii*, but differs by the whole plant being white villous (not pubescent), and inflorescence bracts spathulate and villous (not obovate-spatulate with apex emarginate and sparsely pubescent).

Perennial herbs, 20−35 cm tall. Stems cylindrical or sometimes quadrangular, base decumbent and usually rooting at nodes then erect, densely white villous. Leaves opposite; petiole 0.8−1.2 cm long, villous; blades ovate, oval to sometimes lanceolate, 3.5−7.5 × 1.5−4 cm, papery, apex shortly caudate or acute, margin usually entire or slightly undulate, base cuneate to shortly attenuate, both surfaces densely white villous, mid-vein and secondary veins prominent on both surfaces, secondary veins (5 or) 6 on each side of mid-vein, covered with grayish white strip-like cystoliths. Spikes axillary, ca. 1 cm, usually several flowers in a cluster. Inflorescence bracts leaf-like, usually spathulate, rarely obovate, 7−8.5 × 4−5 mm, with a petiole 3−5 mm long, villous, pinnately veined with white strip-like cystoliths, apex round or sometimes obtuse, base decurrent onto petiole. Bracts and bracteoles linear, 1−1.2 mm long, white villous relatively sparsely below the middle. Calyx 7−9 mm, 5-lobed almost to base; lobes linear-lanceolate with conspicuous membranous margins, white villous, especially above the middle, apex acuminate. Corolla ca. 9 mm long, outside white villous, tube and upper lip white, lower lip white with purple spots; tube slightly longer than limb; upper lip triangular, minutely 2-lobed; lower lip 3-lobed, lobes imbricate, suborbicular. Stamens 2, attached to corolla tube, exserted; filaments ca. 2.2 cm long, basally villous; anther bithecous, thecae superposed, upper one smaller and muticous, lower one larger and with a white spur at base, anther connective pubescent. Ovary glabrous, green, fusiform with nectary flower disc bowl-shaped at base; style ca. 5 mm long, sparsely villous at base, stigma slightly 2-lobed. Capsule fusiform, 6−7 mm, glabrous, sometimes pilose at the tip. Seeds 4, compressed, ca. 1.1 × 1 mm, somewhat heart-shaped or obovate, brown or yellowish-brown, testa tuberculate.

**Figure 1.**
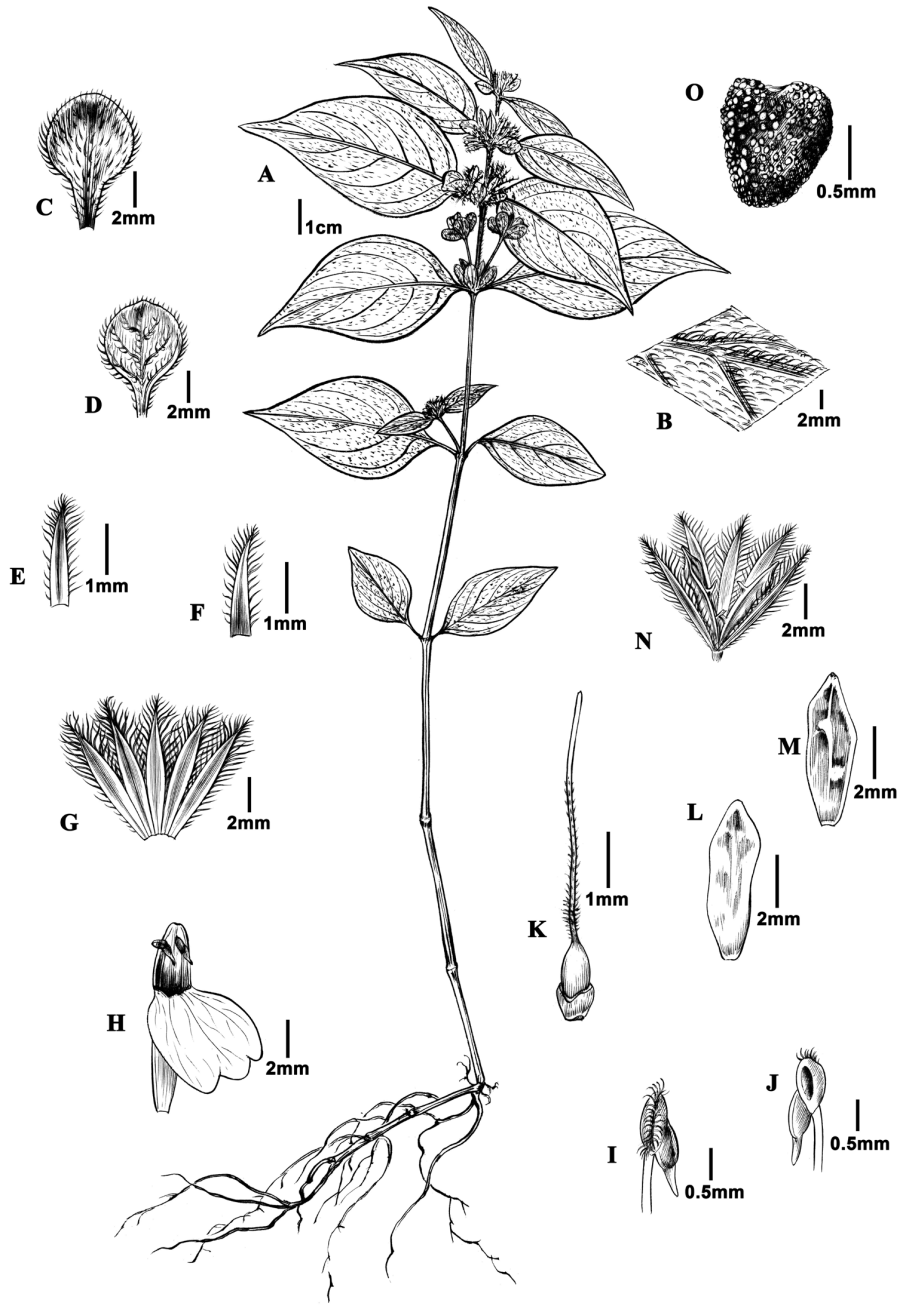
*Justiciathailandica***A** habit **B** magnifying the portion of leaf blades showing the tomentum **C** adaxil surface of inflorescence bract **D** abaxil surface of inflorescence bract **E** bract **F** bracteoles **G** calyx **H** corolla **I** dorsal view of the anther **J** frontal view of the anthers **K** pistil with nectary disc **L**, **M** opened capsule **N** capsule with calyx **O** seed. (Drawn by Cui Dinghan from the holotype van Beusekom et al. 3759).

**Figure 2.**
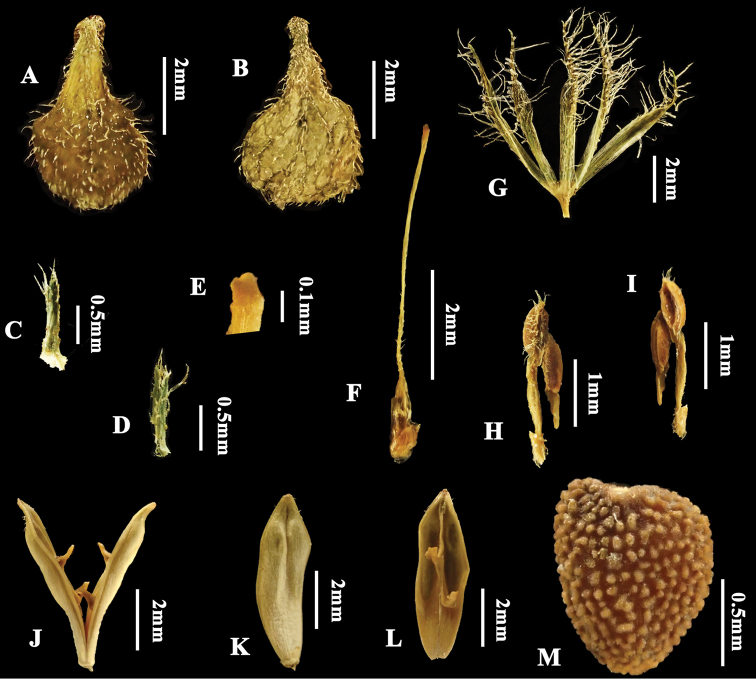
*Justiciathailandica***A** adaxial surface of inflorescence bract **B** abaxial surface of inflorescence bract **C** bract **D** bracteoles **E** stigma **F** pistil with nectary disc **G** calyx **H** dorsal view of anther **I** frontal view of anther **J** opened capsule **K** outside of capsule **L** inside of capsule showing retinacula **M** seed.

#### Etymology.

The specific epithet “*thailandica*” is derived from Thailand, where the new species is found.

#### Phenology.

The new species was recorded in flowering from August to November and fruiting from October to December.

#### Distribution and habitat.

*Justiciathailandica* is only known from Thailand (Fig. 4). It grows in the thickets on the limestone hills or evergreen forest at elevations of 400−900 m.

#### Conservation status.

Currently, *Justiciathailandica* is only known from seven locations of Thailand with eight collections and the estimated extent of occurrence more than 20000 km^2^. We speculate that it may be widespread in Thailand, but is likely to qualify for a threatened category in the near future due to its vulnerable karst habitat and human activities leading to a decline in such habitats. It is therefore assessed as “Near threatened” (NE) according to the IUCN Red List categories and criteria (IUCN 2017).

#### Additional specimens examined.

**THAILAND**. **Chiang Mai**: Chiang Dao District, Doi Chiang Dao, SE foothills near Ban Yang Pong Luang, 575 m alt., 30 Sep 1989, J. F. Maxwell 89-1169 (A, CAS, L). **Kanchanaburi**: Thong Pha Phum District, Krieng Kwia, 420 m alt., 27 Nov 1982, H. Koyama, H. Terao & T. Wongprasert 30402 (BKF, C, K). **Khon Kaen**: Phu Khieo Game Reserve, ca. 80 km east of Phetchabun, 16°50'00"N, 101°58'00"E, 850 m alt., 8 Nov 1984, G. Murata, C. Phengklai, S. Mitsuta, T. Yahara, H. Nagumasu & N. Nantasan T-41809 (A, BKF, TI). **Loei**: Nam Nao National Park, 101°23'00"−28'00"N, 16°48'00"−49'00"E, near check point of road to National Park, 280−350 m alt., 28 Oct 1984, Gen Murata, C. Phengklai, S. Mitsuta, T. Yaahara, G. Nagamasu & N. Nantasan T-51534 (TI); Pha Som Dej-Phataalern, Phu Luang NP., 1000 m alt., 14 Oct 2000, M. Norsaengsri 1075 (QBG). **Nakhon Ratchasima**: Pak Thong Chai District, Salika Forest, 40 km SE from Pak Thong Chai, 14°40'00"N, 102°2'00"E, 400 m alt., 25 Oct 1971, C. F. van Beusekom, Chan Wid & R. Geesink 3362 (BKF, C, K, L, MO, P). **Phetchabun**: Nam Nao District, Nam Nao National Park, 25 Dec 1982, H. Koyama, H. Terao & T. Wongprasert T-31662 (BKF); Nam Nao, 24 Aug 2006, P. Chantaranothai et al. s.n. (BKF, QBG); Loam Gow District, Nahaw Now, 900 m alt., 17 Nov 1973, J. F. Maxwell 73-614 (AAU).

##### Pollen and seed morphology

**Figure 3.**
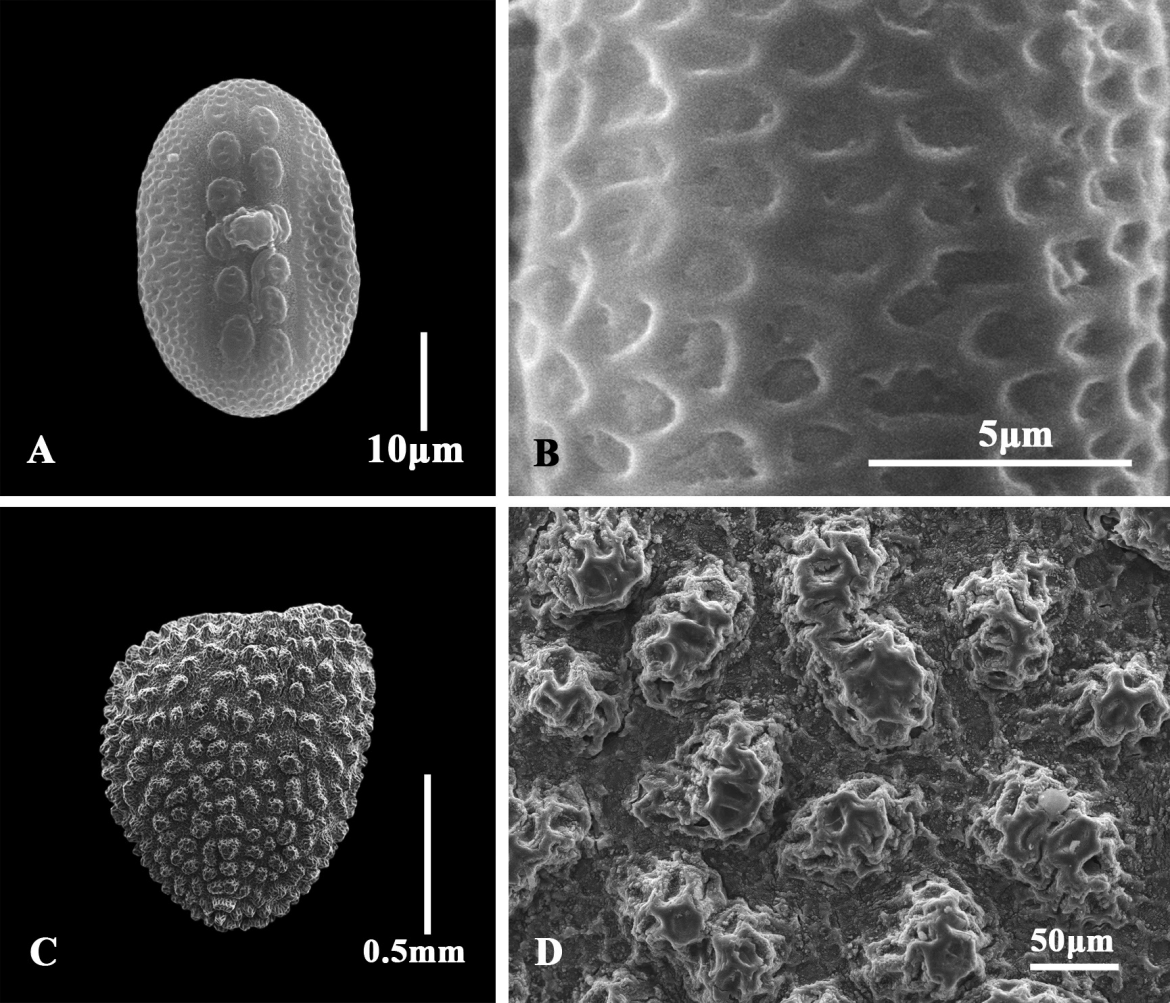
Pollen and seed morphology of *Justiciathailandica* under SEM **A** pollen grain in equatorial view **B** exine ornamentation of pollen grain **C** seed **D** seed testa.

Pollen grains of *Justiciathailandica* are 2-colporate, bilaterally symmetrical, elliptic in both polar and equatorial view, polar axis [P]=33.0±1.55 μm, equatorial diameter [E]=21.6±0.85 μm, P/E=1.52±0.06, aperture area with 2 rows of 6−7 unequal-sized insulae, ornamentation of the insulae microreticulate with few scattered granules between muri (Fig. 3: A–B).

Seeds of *Justiciathailandica* are compressed, somewhat heart-shaped or obovate, brown or yellowish-brown, 1.1−1.2 × 1−1.1 mm, testa densely tuberculate with conspicuous rounded or oblong tubercles, ornamentation of tubercles irregular polygonal (Fig. 3: C–D).

**Figure 4.**
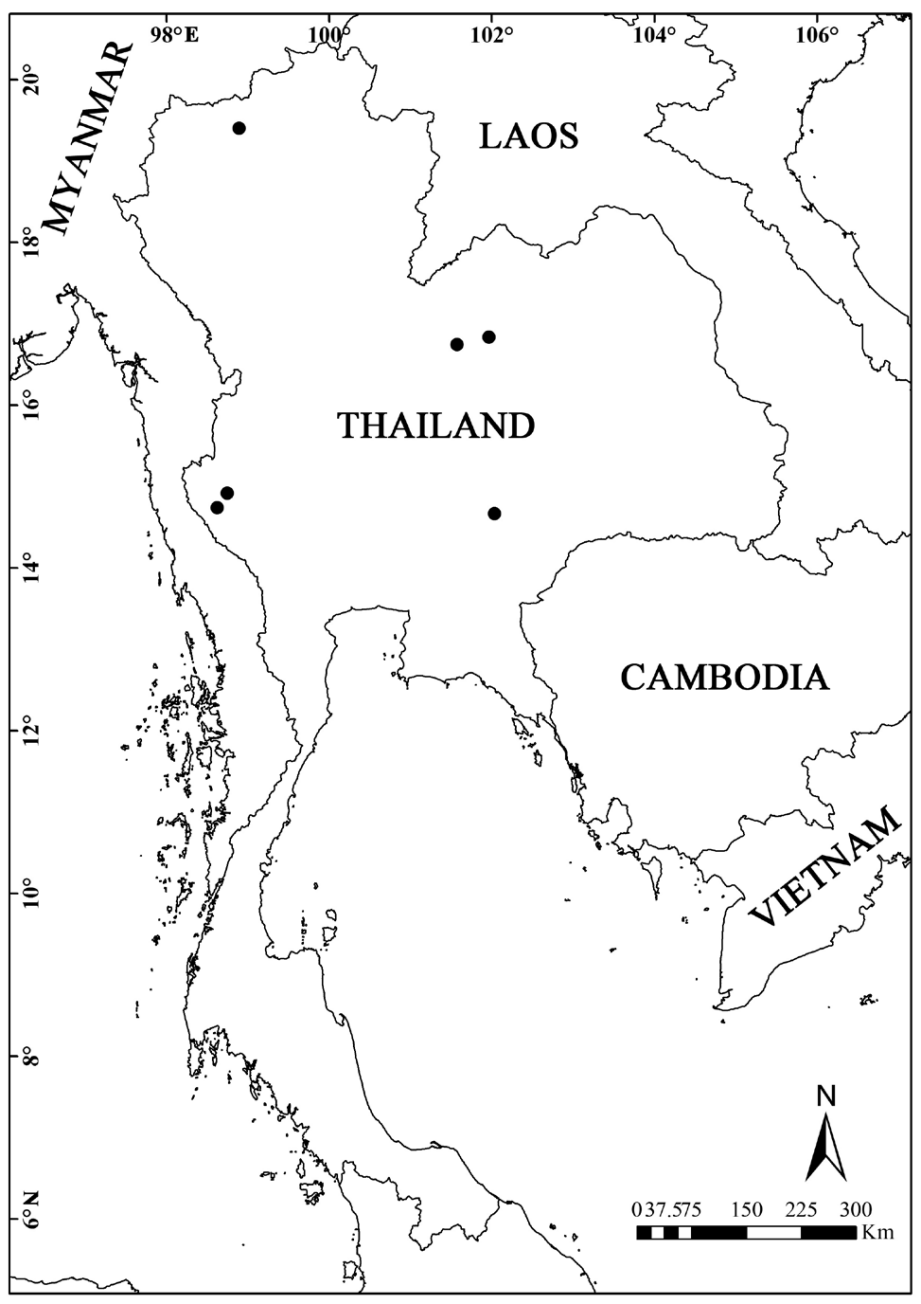
Distribution map of *Justiciathailandica* (black circle).

## Discussion

Characters of pollen grains and seed testa have proved valuable in sectional delimitation in the genus *Justicia* (Graham 1988). The pollen grains of Justiciasect.Harnieria are 2-colporate, reticulate exine ornamentation and traversed by 2 rows of unequal-sized insulae (Graham 1988; Hedrén 1989; Hu et al. 2005; Rueangsawang et al. 2013; Tong et al. 2016). Seed testa of Justiciasect.Harnieria is characterized by rugulose-tuberculate with the apices of the projections pointed (Graham 1988; Hedrén 1989; Rueangsawang et al. 2012; Tong et al. 2016), i.e. “Rugulose-tuberculate” type of Graham (1988). Our observation of the pollen and seed morphology (Fig. 3: A–D) is consistent with that of Justiciasect.Harnieria (Graham 1988; Hu et al. 2005; Hedrén 1989; Rueangsawang et al. 2012, 2013; Tong et al. 2016) and due to the characters of the abbreviated axillary spikes, leaf-like inflorescence bracts, fusiform capsules, we place the new species into that section.

Only three species of Justiciasect.Harnieria have been reported from Thailand. Hosseus (1908) reported *J.quadrifaria* from Thailand based on specimen “Hosseus et al. 228”. Subsequently, Imlay (1938) added J.quardrifariavar.salicifolia (T. Anderson) Imlay based on collections “Marcan 1528 and Lakshnakara 772”. Rueangsawang (2012) and Rueangsawang et al. (2012) recorded three species based on several specimens quoted in text, viz. *J.quardrifaria*, *J.neesiana* (Nees) T. Anderson and *J.championii*.

**Figure 5.**
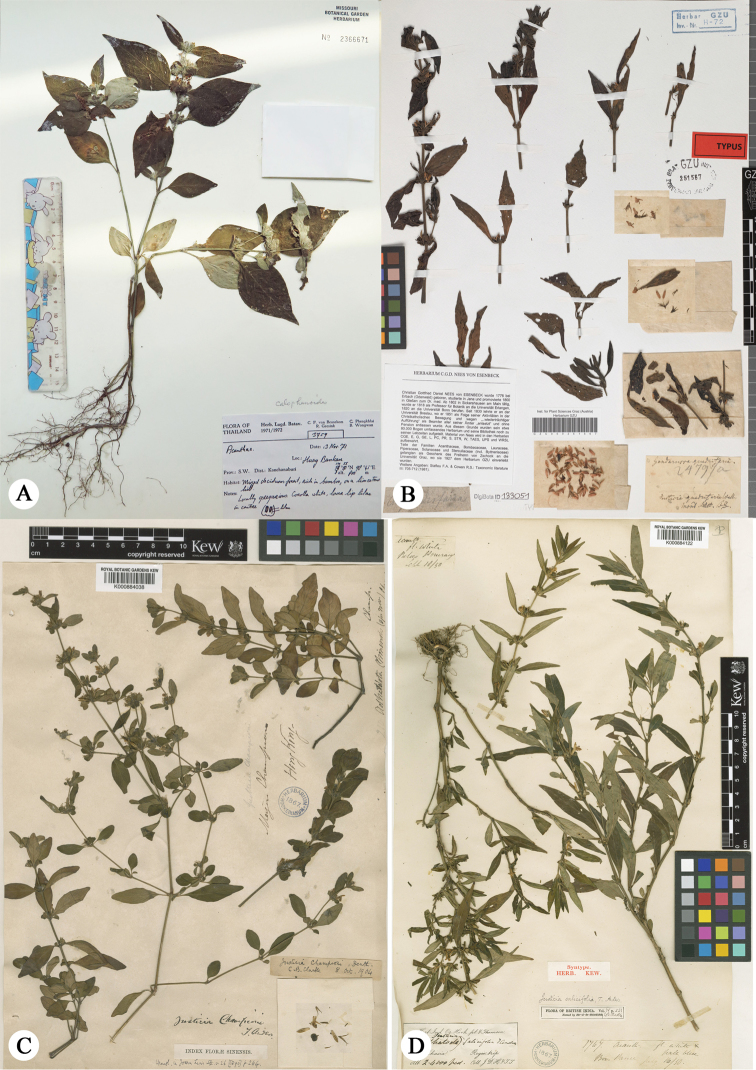
Comparison between *Justiciathailandica*, *J.quadrifaria*, *J.championii* and *J.salicifolia***A** holotype of *Justiciathailandica* (Beusekom *et al.* 3759, MO2366671) **B** isotype of *J.quadrifaria* (Wallich 2479a, GZU000251567) **C** holotype of *J.championii* (Champion 210, K000884038) **D** lectotype of *Justiciabarapaniensis* P. Soumya & Sunojk. (a new name (Soumya 2017) for *J.salicifolia* T. Anderson). Hooker & Thomson s.n., K000884122.

*Justiciachampionii* was first recorded in Thailand by Rueangsawang (2012) based on specimens (e.g. Wongprasert et al. 30402, Maxwell 73–614). However, *J.championii*, is currently known only from China and N Vietnam according to our worldwide specimen examination and is very similar to *J.quadrifaria*, but differs by the leaf shape and may be merged with the latter. After detailed comparison between the type specimen of *J.championii* and some Thai specimens (e.g. van Beusekom et al. 3759, Koyama et al. 30402, van Beusekom et al. 3362) identified in herbaria as *J.championii* or *J.quadrifaria*, we found they are obviously different and represent the new species described above. The clearest difference between *J.thailandica* and *J.championii* is that the calyx is densely white villous in *J.thailandica* (not pubescent) and inflorescence bracts are spathulate and villous in *J.thailandica* (not obovate with apex emarginate and sparsely pubescent). The leaves also tend to be different with the leaf blade ovate and white villous in *J.thailandica* (not lanceolate, oblong to oval and pubescent) with margin usually entire (not slightly undulate), apex shortly caudate or acute (not obtuse), base cuneate (not decurrent), (Fig. 5; Tab. 1). However, these leaf differences represent trends in a spectrum of variation rather than clear discontinuities.

**Table 1.** Comparison of *Justiciathailandica*, *J.quadrifaria*, *J.championii* and *J.zollingeriana*.

	*** Justicia thailandica ***	*** J. quadrifaria ***	*** J. championii ***	*** J. zollingeriana ***
**Opposite leaf**	equal or subequal	equal or subequal	equal or subequal	obviously unequal
**Leaf size**	3.5-7.5 × 1.5−4 cm	1-2 × 5.5-6.5 cm	1–7(–10.5) × 0.5– 2(–3.5) cm	5–10 × 2–3.5 cm
**Leaf shape**	ovate, oval to sometimes lanceolate with margin entire, slightly undulate	oval, oblong to rarely ovate with margin slightly undulate	lanceolate, oblong to oval and pubescent with margin slightly undulate	oblong to lanceolate with margin slightly undulate
**Leaf apex**	shortly caudate or acute	acuminate	obtuse	acuminate
**Leaf base**	cuneate or shortly attenuate	decurrent onto the petiole	decurrent onto the petiole	cuneate
**Petiole length**	8−12 mm	5−7 mm	5–15 mm	7−15 mm
**Lateral leaf vein**	(5)6	7(8)	6(7)	5(6)
**Indumentum**	densely white villous	pubescent	densely pubescent	glabrous
**Inflorescence bracts**	spathulate and densely villous	ovate to obovate and sparsely pubescent	obovate-spatulate with apex emarginate and sparsely pubescent	spathulate to round and glabrous
**Calyx**	7–9 mm, densely white long villous	7–7.3 mm, pubescent	7–9.5 mm, pubescent	4–5 mm, glabrous
**Calyx-capsule length ratio**	0.7–0.82	1.07–1.32	0.89–1.06	0.59–0.67
**Flowering**	Aug–Nov.	Unknown	Aug–Oct.	Jul-Sep.
**Fruiting**	Oct–Dec.	Unknown	Aug–Oct.	Jul-Sep.
**Distribution**	Thailand	India and Bangladesh	South China and north Vietnam	From Thai Peninsular to Malay Archipelago

Morphologically, *Justiciathailandica* is also similar to *J.quadrifaria*, but it can be easily distinguished from *J.quadrifaria* by the leaf blade being ovate and white villous (not oval, oblong to rarely ovate and pubescent), margin entire (not slightly undulate), petiole 8−12 mm (not 5−7 mm) long, apex shortly caudate or acute (not acuminate), base cuneate (not decurrent onto the petiole), calyx densely white villous (not pubescent), inflorescence bract spathulate and villous (not ovate to obovate and sparsely pubescent) and mature capsule longer than the calyx (not shorter than the calyx).

*Justiciaquadrifaria* was recognized to be widely distributed in the tropical region of S to SE Asia from India, Indochina to Malay Archipelago and South China (Ridley (1923; Hu et al. 2011). However, it is restricted to NE India and Bangladesh based on our worldwide specimen examination. *J.zollingeriana* (Nees) C. B. Clarke was reduced to *J.quadrifaria* or *Calophnodesquadrifaria* by some authors (Clarke 1907; Ridley 1923, Hu et al. 2011). However, it can be easily distinguished from the latter by the plant being glabrous (not pubescent in *J.quadrifaria*), calyx 4–5 mm (not 7–7.3 mm), capsule obviously more than 1.5 times longer than the calyx (not shorter than or subequal to the calyx) and leaves in each pair obviously unequal in size (not equal or subequal). *J.zollingeriana* is distributed in SE Asia from Thai Peninsular to Malay Archipelago. In Thailand, the species was first recorded as a synonym of *J.quadrifaria* by Hosseus (1908) based on specimen “Hosseus et al. 228”, and then some specimen (e.g. Kerr 7375, Garrett 316) of the species were reported under *J.quadrifaria* by Imlay (1938), while some specimen (Marcan 1528 and Lakshnakara 772) were under J.quadrifariavar.salicifolia by Imlay (1938).

*Justicianeesiana* recorded by Rueangsawang (2012) and Rueangsawang et al. (2012) is very similar to *J.multinodis* in the lanceolate leaves, however, it differs from the latter by the plant being pubescent (not nearly glabrous in later), leaf 39–47 × 5–7.5 mm (not 22–37 × 2.2–4 mm), base decurrent onto the petiole (not cuneate), lateral leaf vein 5 with veinlet not reticulate (not usually 7 with veinlet obviously reticulate), petiole 5–6 mm (not nearly sessile), axillary spikes usually with 3–5 flowers (not 2–3 flowers) and inflorescence bracts subrotund to oval, persistent (not lanceolate, caducous).

In the course of revising Thailand species of sect.Harnieria, we have confirmed there are three species in Thailand, viz. *J.zollingeriana* (Nees) C. B. Clarke, *J.multinodis* R. Benoist and a new species, *J.thailandica*, described here.

A comparison of characters between *Justiciathailandica*, *J.quadrifaria*, *J.championii* and *J.zollingeriana* is provided in Table 1. An identification key to Thai species in sect.Harnieria is provided below.

### Identification key to Thai species in Justiciasect.Harnieria

**Table:** 

1	Mature capsule more than 1.5 times longer than the calyx in length, leaves in each pair obviously unequal in size	*** Justicia zollingeriana ***
–	Mature capsule short than or sub-equal to the calyx, leaves in each pair subequal in size	**2**
2	Inflorescence bract and calyx lobes obviously with dense long villous hairs; leaf large, ovate, 3.5−7.5 × 1.5−4 cm	*** J. thailandica ***
–	Inflorescence bract and calyx lobes nearly glabrous or with sparsely pubescent hairs; leaf small, narrowly lanceolate, 3−7 × 0.6−0.9 cm	*** J. multinodis ***

## Supplementary Material

XML Treatment for Justicia thailandica
